# Supplementary material for: Species-Specific N-Glycomes and Methylation Patterns of Oysters Crassostrea gigas and Ostrea edulis and Their Possible Consequences for the Norovirus–HBGA Interaction
Source: Mar Drugs. 2023 Jun 2;21(6):342. doi: 10.3390/md21060342 (PMC10301044; doi:10.3390/md21060342)
Supplement: Supplementary file 1 [file marinedrugs-21-00342-s001.zip › marinedrugs-2413677-supplementary.pdf]

Supplementary material

Species-Specific *N*-Glycomes and Methylation Patterns of Oysters *Crassostrea gigas* and *Ostrea edulis* and Their Possible Consequences for the Norovirus–HBGA Interaction

Figure S1. Monosaccharide composition analysis of *N*-glycans. Total Ion Count generated from GC-MS analysis of reduced and permethylated derivatives of (A) authentic standards, (B) *N*-glycans released from the mantle of *C. gigas* by PNGase F, (C) *N*-glycans released from the mantle of *O. edulis* by PNGase F.

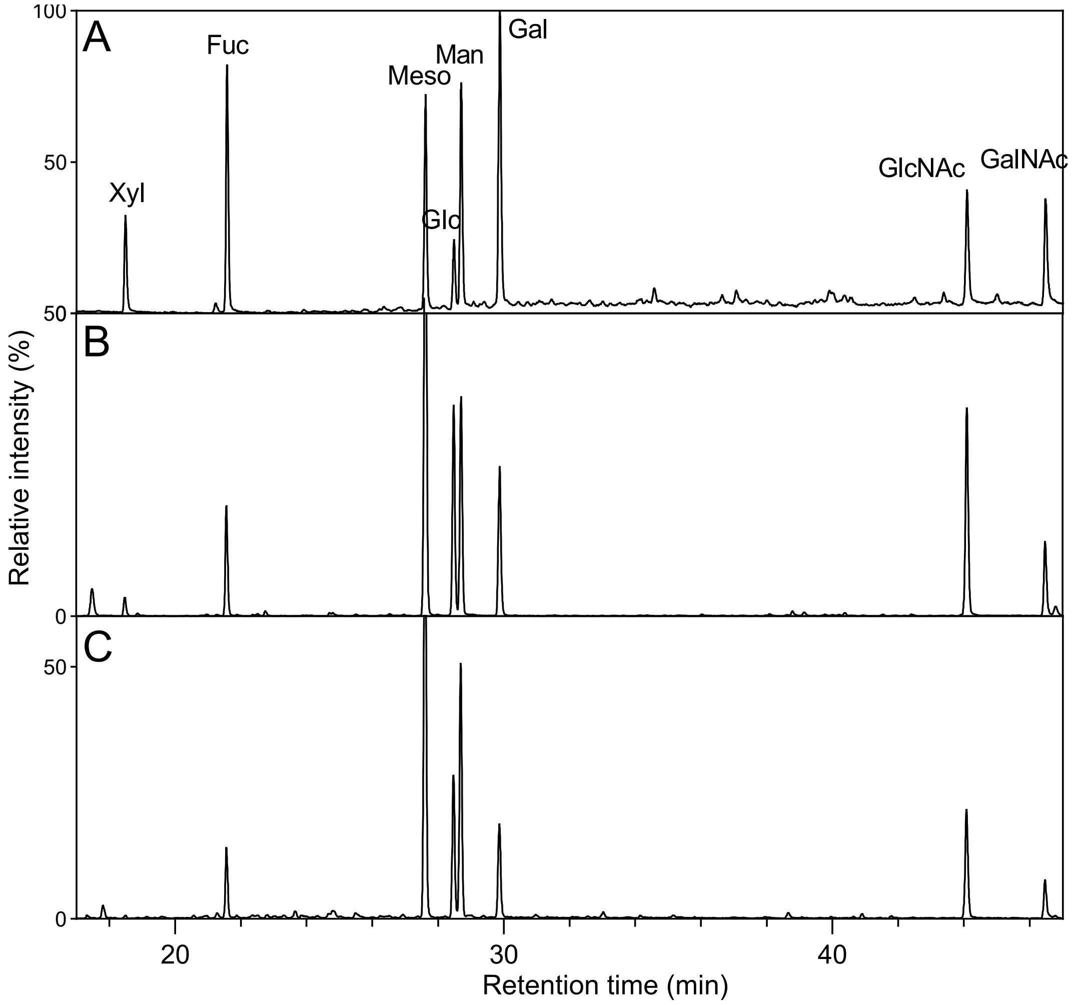

Figure S2. Ratios (in %) of Me-3*O*-Man (light green) and Me-4*O*-Man (dark green) in *N*-glycans isolated from individual tissues of *C. gigas* and *O. edulis* (G, gills; D, digestive tissue; M, mantle) using (A) PNGase F and (B) PNGase A.

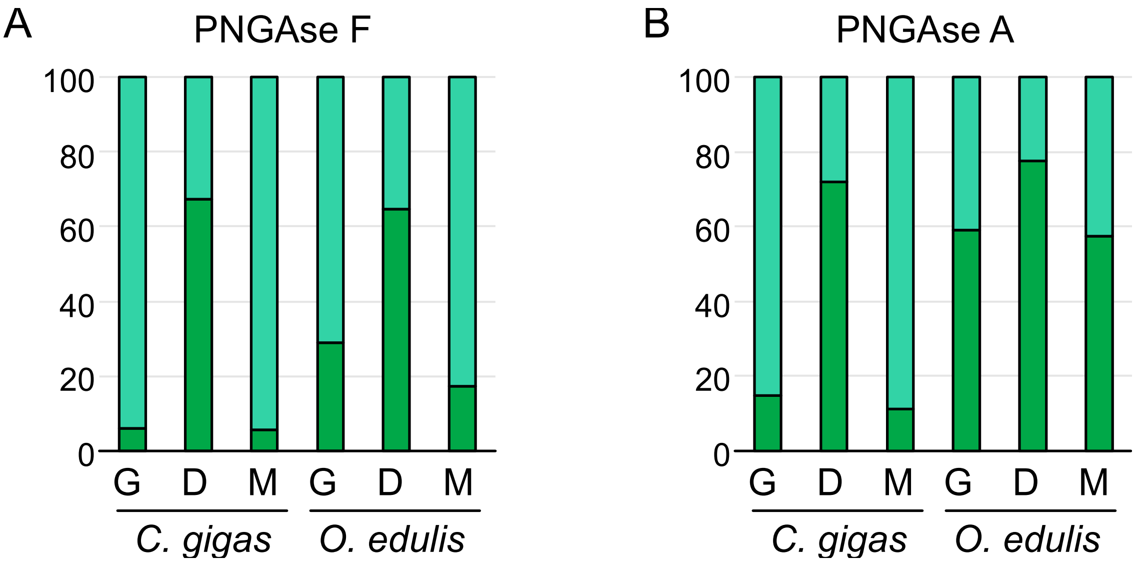

Table S1. Quantification of monosaccharides in *N*-glycans sequentially released by PNGase A and F of (**A**) *Crassostrea gigas* (Cg) and **(B)** *Ostrea edulis* (Oe) in the three analysed organs gills (G), digestive track (D) and mantle (M). Values are expressed in ng of monosaccharides per mg of proteins.

A

	PNGAse F			PNGAse A			Total PNGase A + F
	Gg-G	Cg-D	Cg-M	Cg-G	Cg-D	Cg-M	
MeFuc	27,8	28,7	20,0	0,5	6,5	3,6	87,0
Fuc	37,4	115,5	71,2	12,9	52,3	36,2	325,6
Xyl	3,1	15,1	13,1	3,4	2,9	7,8	45,5
MeMan	5,0	78,7	33,9	0,7	15,6	10,7	144,6
MeGal	1,0	1,3	0,6	0,0	0,6	0,3	3,8
Man	20,2	227,1	105,7	3,5	28,9	25,2	410,5
Gal	36,5	64,1	47,5	10,9	15,3	14,5	188,7
MeGalNAc	6,1	14,2	15,9	0,8	1,5	3,6	42,2
GlcNAc	18,8	141,8	71,7	2,9	26,1	21,6	282,9
GalNAc	5,4	6,8	4,2	0,6	1,0	0,9	19,0
Total	161,2	693,2	383,8	36,3	150,8	124,5	1549,8

B

	PNGAse F			PNGAse A			Total PNGase A + F
	Oe-G	Oe-D	Oe-M	Oe-G	Oe-D	Oe-M	
MeFuc	162,0	71,6	133,7	15,7	5,9	18,6	407,6
Fuc	313,2	221,6	256,3	62,1	36,2	53,2	942,6
Xyl	4,8	5,0	6,7	4,2	2,9	3,6	27,2
MeMan	194,3	113,8	196,6	25,8	10,2	18,3	558,9
MeGal	28,4	12,5	10,7	4,4	2,2	5,0	63,3
Man	510,9	365,1	494,6	46,1	21,2	38,0	1475,9
Gal	253,8	150,6	166,9	29,6	23,2	36,4	660,4
MeGalNAc	70,7	25,3	70,6	5,0	2,5	8,5	182,7
GlcNAc	346,6	212,8	366,9	44,5	20,8	46,6	1038,1
GalNAc	31,0	12,9	20,7	2,1	2,0	4,5	73,2
Total	1915,7	1191,1	1723,9	239,4	127,1	232,7	5429,9

Table S2. List of *N*-glycans identified as permethylated derivatives following sequential released by PNGases F and PNGase A from three tissues gills (G), digestive tract (D) and mantle (M) of two oyster species, *Crassostrea gigas* (CG) and *Ostrea edulis* (OE). Structures were deduced from MS and MS^n analyses of permethyl derivatives. *N*-glycans are ordered in increasing molecular weight. The presence of different *N*-glycans in each organ is indicated by a blue rectangle for *Crassostrea gigas* and green for *Ostrea edulis,* irrespective of the presence of natural methyl groups. Structures were represented according the standard Symbol Nomenclature for Glycan system [1].

name	Theorical m/z	Structure	*C.gigas*			*O.edulis*		
			G	D	M	G	D	M
CG1, OE1	1141,57	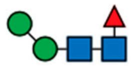						
CG2, OE2	1141,57	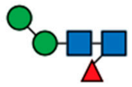						
CG3, OE3	1171,58	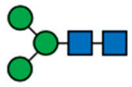						
CG4	1301,65	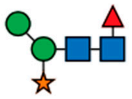						
CG5, OE4	1301,65	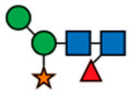						
CG6, OE5	1315,66	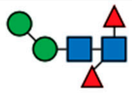						
CG7, OE6	1331,66	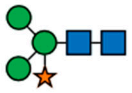						
OE7	1331,66	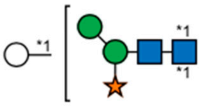						
CG8, OE8	1345,67	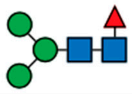						
CG9, OE9	1345,67	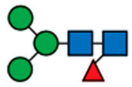						
CG10	1345,67	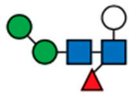						
OE10	1345,67	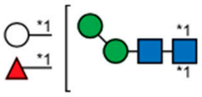						
CG11, OE11	1375,68	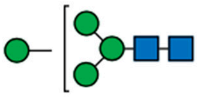						
OE12	1375,68	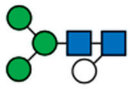						
OE13	1375,68	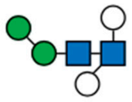						
CG12	1416,71	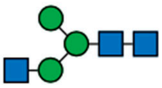						

CG13, OE14	1475,74	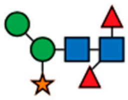						
CG14	1505,75	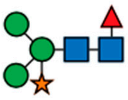						
CG15, OE15	1505,75	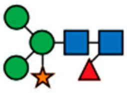						
CG16, OE16	1505,75	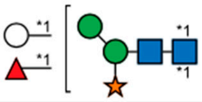						
CG17, OE17	1519,76	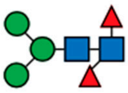						
CG18, OE18	1535,76	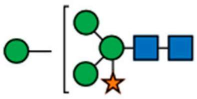						
OE19	1535,76	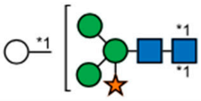						
OE20	1535,76	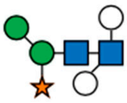						
CG19	1549,77	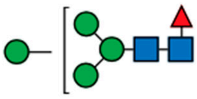						
CG20, OE21	1549,77	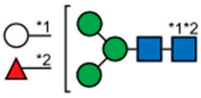						
CG21, OE22	1549,77	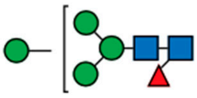						
CG22, OE23	1549,77	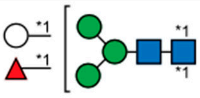						
CG23	1576,78	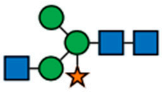						
CG24, OE24	1579,78	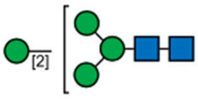						
CG25	1590,8	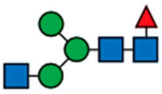						
CG26	1590,8	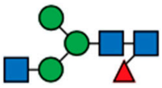						

CG27	1620,81	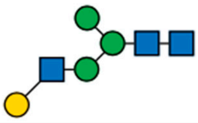
CG28, OE25	1679,84	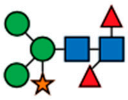
CG29	1709,85	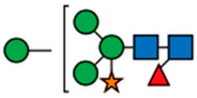
CG30	1709,85	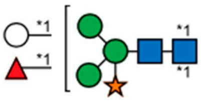
CG31	1723,86	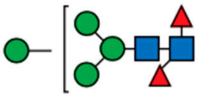
CG32	1723,86	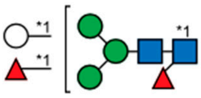
CG33	1750,87	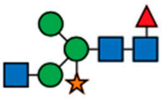
CG34	1750,87	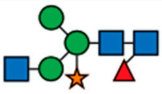
CG35, OE26	1753,87	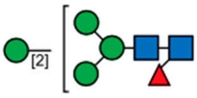
CG36	1764,89	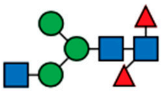
CG37	1780,88	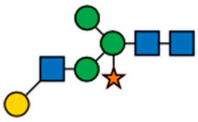
CG38, OE27	1783,88	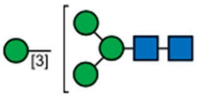
CG39	1794,9	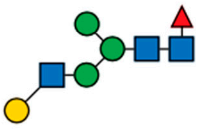
CG40	1794,9	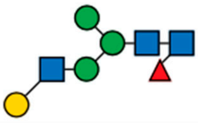
OE28	1794,9	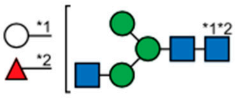

OE29	1794,9	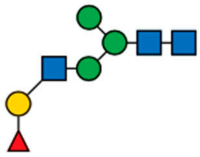
OE30	1794,9	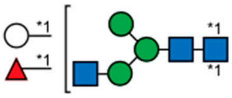
CG41	1824,91	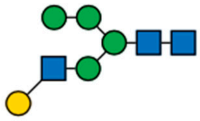
CG42	1913,95	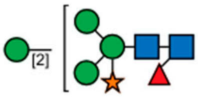
CG43	1924,96	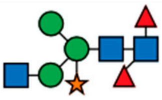
CG44	1954,97	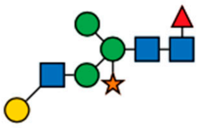
CG45	1954,97	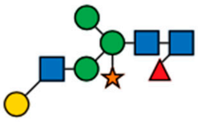
CG46	1968,99	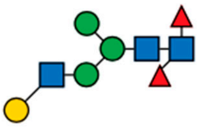
OE31	1968,99	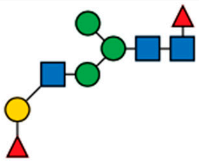
CG47, OE32	1987,98	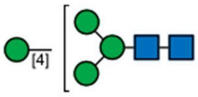
CG48, OE33	2040,02	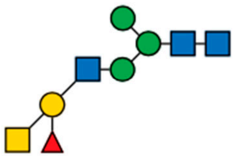
CG49	2070,03	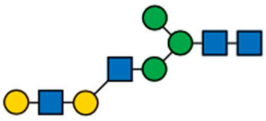
CG50	2118,05	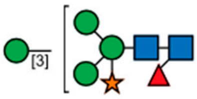

CG51	2129,06	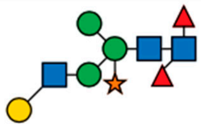						
OE34	2143,08	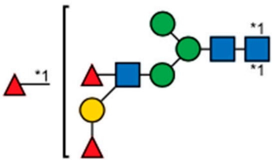						
OE35	2143,08	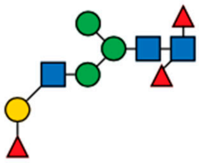						
OE36	2173,09	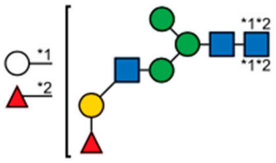						
OE37	2173,09	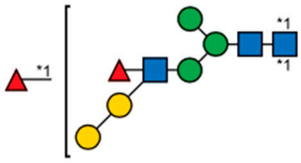						
CG52, OE38	2192,08	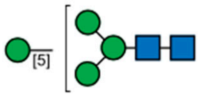						
CG53	2200,1	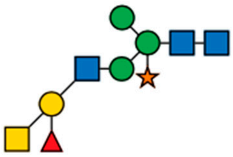						
CG54, OE39	2214,11	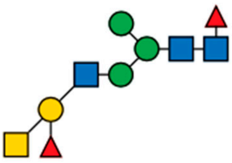						
CG55	2214,11	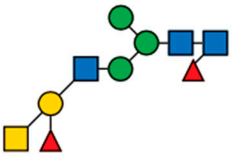						
CG56	2230,11	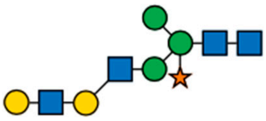						
CG57, OE40	2244,12	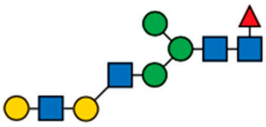						

CG58	2244,12	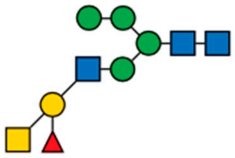
OE41	2244,12	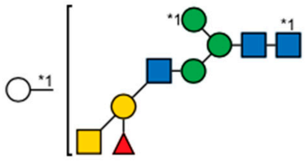
CG59	2285,15	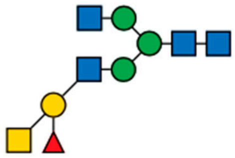
OE42	2347,18	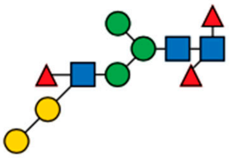
CG60	2374,19	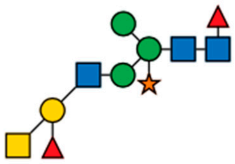
OE43	2377,19	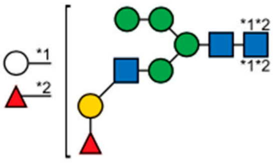
CG61, OE44	2388,2	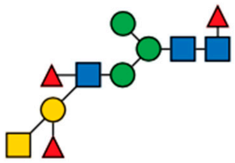
CG62, OE45	2388,2	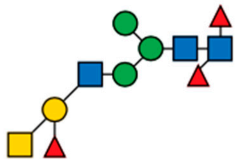
CG63, OE46	2396,18	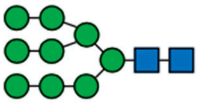
CG64	2404,2	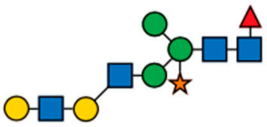

CG65, OE47	2418,21	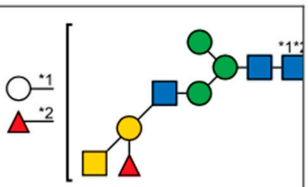
CG66	2418,21	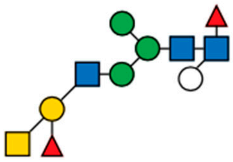
CG67	2445,22	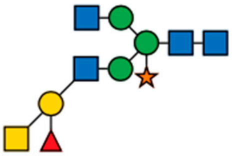
CG68	2459,24	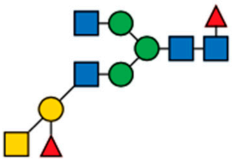
CG69	2489,25	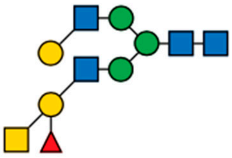
CG70	2548,28	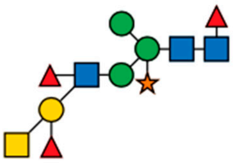
CG71, OE48	2548,28	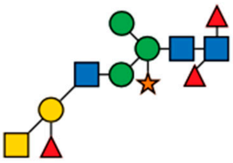
CG72, OE49	2562,29	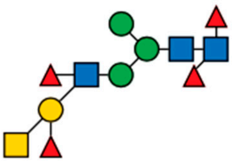
CG73	2578,29	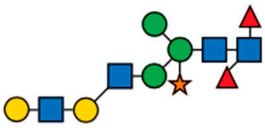
CG74, OE50	2600,3	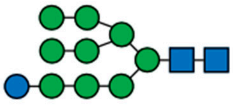

CG75	2649,32	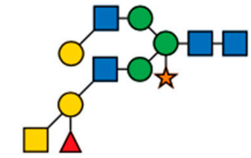						
CG76, OE51	2663,34	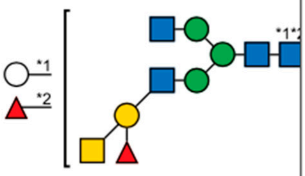						
CG77	2722,37	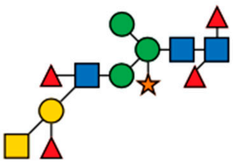						
CG78	2793,4	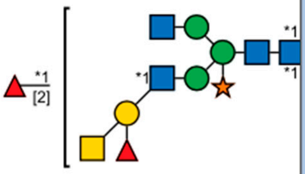						
OE52	2837,43	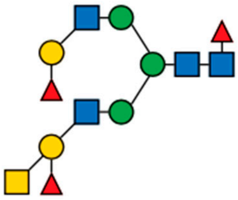						
CG79, OE53	2908,47	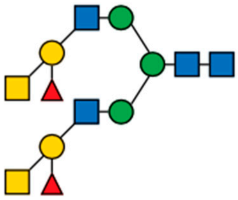						
CG80	2938,48	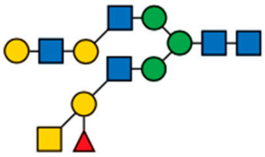						
CG81	3068,54	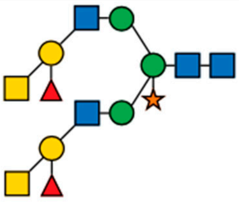						
CG82, OE54	3082,55	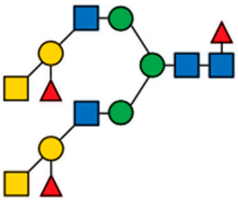						

CG83	3098,55	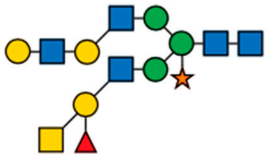
OE55	3112,57	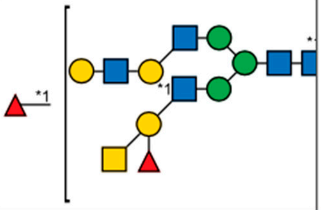
CG84	3242,63	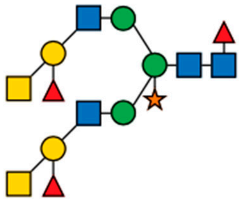
CG85, OE56	3256,64	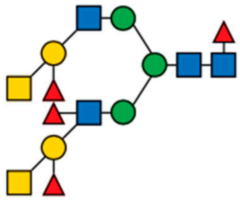
OE57	3286,66	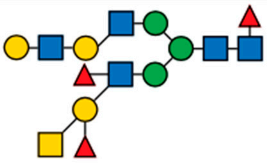
CG86	3416,72	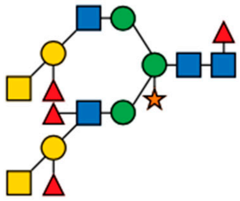
CG87, OE58	3430,73	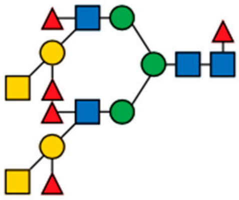
CG88	3590,81	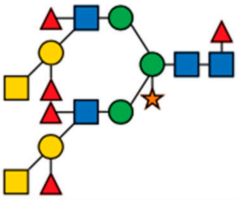

OE59	3951	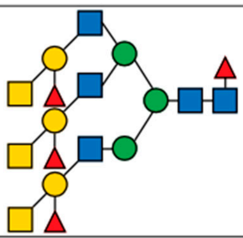						

Table S3. List of methylated N-glycans identified from the mantle of *Crassostrea gigas* and *Ostrea edulis*. Following their sequential release from *C.gigas* and *O.edulis* by PNGase F and A, *N*-glycans were deuteromethylated and analysed by MALDI-QIT-TOF. Each -3 m.u. difference between the expected mass and the measured mass indicated of the presence of a methyl group in the native *N*-glycan. The number of methyl groups per *N*-glycan is indicated by the presence of a cross in the corresponding column of the table (yellow for *c.gigas* and blue for *O.edulis*).

N-glycan	0	1	2	3
CG1		x		
CG2	x	x		
CG3	x	x	x	
CG4		x		
CG5		x		
CG6	x	x		
CG7	x	x	x	
CG8		x	x	
CG9	x	x	x	
CG10	x	x		
CG11	x	x	x	
CG12		x		
CG13		x		
CG14	x	x	x	
CG15		x	x	
CG16		x		
CG17	x	x	x	
CG18		x	x	
CG19			x	
CG20			x	

N-glycan	0	1	2	3
CG21	x	x	x	
CG22	x	x	x	
CG24	x	x	x	x
CG25	x	x		
CG27	x	x	x	
CG28		x	x	
CG33	x	x		
CG35			x	
CG37	x	x		
CG38	x	x	x	x
CG39	x	x		
CG44	x	x		
CG45	x	x		
CG46	x			
CG47	x	x	x	x
CG48			x	
CG49			x	
CG51	x			
CG52	x		x	x
CG53		x	x	

N-glycan	0	1	2	3
CG54			x	
CG55			x	
CG56			x	
CG60		x	x	
CG61				x
CG62			x	x
CG63	x	x	x	
CG69		x		
CG70			x	x
CG71		x	x	
CG74	x			
CG77			x	x
CG78			x	x

N-glycan	0	1	2	3	4
OE1		x			
OE2	x	x			
OE3		x	x		
OE5		x			
OE6			x		
OE8			x		
OE9		x	x		
OE10	x	x			
OE11		x	x		
OE12		x			
OE13		x			
OE14		x			
OE17			x		
OE21			x		
OE22		x	x		
OE23		x	x		
OE24	x		x	x	
OE26			x		
OE27			x	x	
OE29			x		
OE31			x	x	
OE33		x	x		
OE34		x	x	x	
OE35				x	
OE36				x	
OE41	x		x		
OE42		x	x	x	
OE52				x	x
OE54	x	x	x		
